# Tracking and Classification of In-Air Hand Gesture Based on Thermal Guided Joint Filter

**DOI:** 10.3390/s17010166

**Published:** 2017-01-17

**Authors:** Seongwan Kim, Yuseok Ban, Sangyoun Lee

**Affiliations:** Department of Electrical and Electronic Engineering, Yonsei University, 50 Yonsei-ro, Seodaemun-gu, Seoul 03722, Korea; knauer@yonsei.ac.kr (S.K.); van@yonsei.ac.kr (Y.B.)

**Keywords:** joint filter, thermal sensor, visual sensor, hand gesture tracking, hand gesture recognition, varying lighting conditions

## Abstract

The research on hand gestures has attracted many image processing-related studies, as it intuitively conveys the intention of a human as it pertains to motional meaning. Various sensors have been used to exploit the advantages of different modalities for the extraction of important information conveyed by the hand gesture of a user. Although many works have focused on learning the benefits of thermal information from thermal cameras, most have focused on face recognition or human body detection, rather than hand gesture recognition. Additionally, the majority of the works that take advantage of multiple modalities (e.g., the combination of a thermal sensor and a visual sensor), usually adopting simple fusion approaches between the two modalities. As both thermal sensors and visual sensors have their own shortcomings and strengths, we propose a novel joint filter-based hand gesture recognition method to simultaneously exploit the strengths and compensate the shortcomings of each. Our study is motivated by the investigation of the mutual supplementation between thermal and visual information in low feature level for the consistent representation of a hand in the presence of varying lighting conditions. Accordingly, our proposed method leverages the thermal sensor’s stability against luminance and the visual sensors textural detail, while complementing the low resolution and halo effect of thermal sensors and the weakness against illumination of visual sensors. A conventional region tracking method and a deep convolutional neural network have been leveraged to track the trajectory of a hand gesture and to recognize the hand gesture, respectively. Our experimental results show stability in recognizing a hand gesture against varying lighting conditions based on the contribution of the joint kernels of spatial adjacency and thermal range similarity.

## 1. Introduction

Recognizing hand gestures has been extensively studied in various research areas, as hand gestures pertain to the understanding of meaningful expressions of motion by humans [1]. Especially, hand gesture recognition in the field of image processing allows many motivating applications, such as sign language recognition, machine interface control, augmented reality, etc. Various sensors have been leveraged to exploit the benefits of different modalities for the extraction of meaningful information conveyed by a user’s hand gesture. Many studies have demonstrated the apparent advantages of using multiple modalities to improve various tasks [2,3,4,5,6,7,8,9,10,11,12,13]. Lately, the diffusion of relatively low-cost thermal sensors (e.g., a portable camera or a smart phone module) has promoted research on the benefits of thermal information.

Still, most of the conventional thermal-related methods [12,13,14,15,16,17] are focused on face recognition. Thermal sensors have the limitation of low resolution, so the methods used face data acquired at short distances, and mostly tackle the issue of varying illumination. Based on the relative merit of radiometrically-calibrated thermal imagery, comprehensive performance analysis on face recognition shows the effectiveness of the fusion between thermal and visual sensors against the illumination issue [18]. Some works have also studied the performance of detecting a human body [9,10,19], but few works have been studied the recognition of hand gestures with the guidance of a thermal sensor. Visual sensors can provide details with high resolution, though they suffer from performance degradation of hand gesture recognition caused by different lighting conditions. Thermal sensors are able to compensate the shortcoming of visual sensors against different lighting conditions, yet thermal information on its own lacks the details of a hand area and the distinction of the edge of a hand. Notably, the existing approaches deal with the two modalities as independent or with simple fusion methods. More investigation is needed to fully exploit the cooperative relation of the two modalities—thermal and visual sensors.

In this paper, we propose a hand gesture recognition method based on a novel feature-based joint filter; namely, a *thermal guided joint filter*. Our approach is motivated by the investigation of the mutual supplementation between thermal and visual information for the consistent description of a hand gesture. Our proposed method effectively tracks the trajectory of a hand, and recognizes the gesture under varying lighting conditions.

Section 2 gives an introduction to the related works. Section 3 describes our thermal-guided joint filter-based hand gesture recognition method in detail. Section 4 and Section 5 present the experimental results followed by a discussion to demonstrate the contribution of our proposed method. Finally, Section 6 provides our conclusions.

## 2. Background

In contrast to the extensive research on visual sensor-based hand gesture recognition, relatively few systems have been reported that take advantage of thermal information. Particularly, few works have proposed the complementary strategy of using thermal and visual information in the feature computing stage. The existing works rather focus on extracting features only from the thermal image, which is robust against illumination and conveys distinguishable clues from those of visual images. Larson et al. [20] proposed a hand gesture tracking method using the temperature trail marked by touching the surface of a panel. This method is effective for gesture interaction using a touch panel, but is not applicable to the gesture interaction of a larger degree of freedom (e.g., in-air hand gesture interface). Zeng et al. [21] used the strength of thermal cameras in varying illumination to verify the pose of a user in the thermal domain for a presentation scenario based on a beam projector. They dealt with the complex patterns projected onto the projection plane using thermal information. Still, thermal information was solely exploited to verify a pose, while the gesture was mainly tracked using visual information. Additionally, the use of the method is limited to scenarios with a projection plane in which the fragments share similar temperature. Appenrodt et al. [22] compared the performances of gesture recognition based on mono-color images, stereo-color images, and thermal images. However, as mentioned in [20], the performance can be unstable because of the difference in the temperature of a hand among test subjects when using region segmentation by a fixed temperature threshold. Hanif et al. [23] tested the simple fusion of thermal and visual information as addition (i.e., weighted summation) to provide an insight into increasing the recognition performance based on the sensor fusion given the registration between the two modalities. Hanif et al. addressed the problem of changing illumination based on sensor fusion, and verified that the fusion-based methods yield better performance over a single modality.

In this paper, we design a joint filter connoting not only the spatial distance relationship between thermal and visual images, but also the range relationship in order to provide a conjunct feature representation of mutual complement between thermal and visual information. We have built a flexible structure combining thermal and visual information with respect to spatial distance and range at the same time. Based on our method, both visual and thermal sensors can be fully exploited, resulting in hand gesture tracking and recognition which operates robustly under changes in illumination. Our proposed method is applicable to the existing hand gesture tracking and recognition methods, as it directly enriches the visual image with the cooperative role of a thermal image.

## 3. Proposed Method

In this chapter, the thermal guided joint filter is presented in detail, and we describe how to benefit from the cooperative relation between the thermal sensor and the visual sensor.

### 3.1. Calibration, Registration, and Synchronization

**Figure 1:**
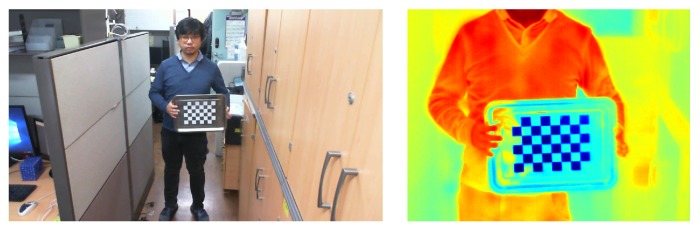
Examples of visual and thermal images used for calibration.

**Figure 2:**
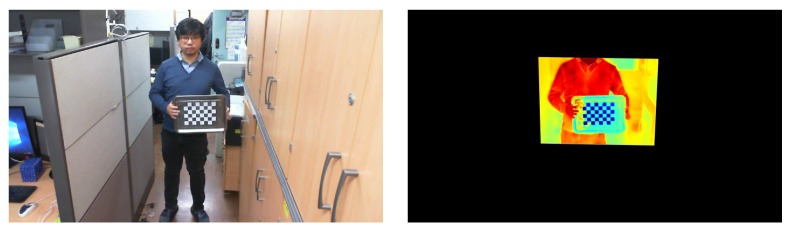
The registration result based on the calibration.

The spatial and temporal correspondence between the thermal image and the visual image are indispensable in order to leverage information from both modalities. First, the adjustment of the spatial difference is obtained by calibration and registration [24,25,26]. The checkerboard for calibration needs to be clearly shown in both the thermal and visual images. Yet, previous works related to thermo-visual calibration need a high production cost for a checkerboard [27], suffer from blurred checkerboard squares [28,29], and are not adoptable to conventional calibration methods [30]. Hence, we suggest a novel type of checkerboard which can be produced at low cost, leads to clear squares, and is compatible with conventional calibration methods. The plate material for the checkerboard is stainless steel, which has high heat conductivity. The squares of the checkerboard in both modalities are clearly presented (as shown in Figure 1) for the following two reasons. First, the large differences in heat conductivity and reflectivity between the stainless steel plate and the white-colored squares made of paper are effective, as we freeze the checkerboard before starting calibration. Second, the intensity levels of the pixels in the thermal image have been normalized for accurate calibration in order to obtain a better registration for the database. Based on the parameters computed from the calibration step, the registration between thermal and visual images is performed as shown in Figure 2. Nevertheless, registration error exists to some extent, and the spatial correspondence of the pixels between thermal and visual images is acquired by the registration step. Second, the adjustment of the temporal difference is obtained by synchronization because the restorations of the two modalities bring about a slight time difference. The video slate by hand clapping has been applied to support the synchronization of thermal and visual images.

### 3.2. Thermal-Guided Joint Filter

**Figure 3:**
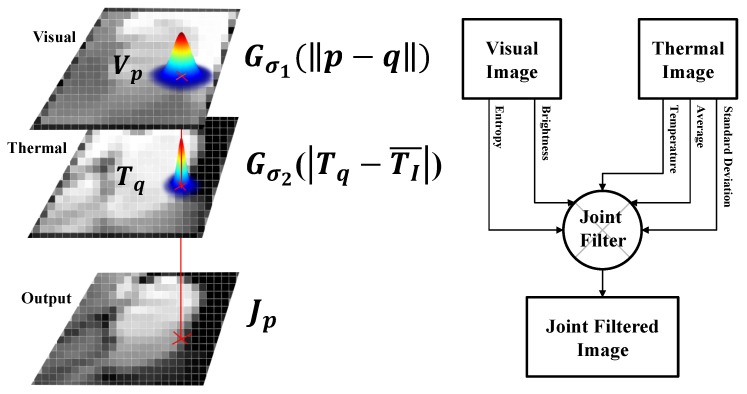
Overall process of our thermal-guided joint filter.

Our proposed joint filter takes advantage of both the brightness information from the visual image and the temperature information from the thermal image. Moreover, the information entropy [31] of brightness from the visual image and the average and standard deviation of temperature from the thermal image are involved in order to adaptively determine the smoothness of the filter by altering brightness and temperature (see Figure 3). We adopt the overall structure from a joint bilateral upsampling filter [32], which includes a spatial kernel and a range kernel. Not only the horizontal and vertical axes, but the range (i.e., intensity) axis also actively takes part in the calculation of the filter for an output.

The joint filtered output at position *p*, $J_{p}$ is computed as in Equation (1), where $V_{p}$ is the visual intensity at position *p*, $T_{q}$ is the thermal temperature at index position *q*, I is the set including all index positions, $\bar{T_{I}}$ is the average temperature from the initial detection region *I*, and *N* is a normalizing factor. $G_{\sigma_{1}}$ and $G_{\sigma_{2}}$ are Gaussian distributions that both have mean values of zero and the standard deviations of $\sigma_{1}$ and $\sigma_{2}$, respectively.

(1) $$J_{p}=N\sum_{q\in I}G_{\sigma_{1}}(||p-q||)G_{\sigma_{2}}\left(T_{q}-\bar{T_{I}}\right)V_{p}$$

$G_{\sigma_{1}}$ is a univariate Gaussian distribution which represents the proposed spatial adjacency kernel (see Equation (2)). $\sigma_{1}$ determines the smoothness of the influence of $G_{\sigma_{1}}$. It is computed by considering the registration error *r* between thermal and visual images and the halo effect *h* of the thermal camera, per Equation (3). The smoothness is obtained by a positive term and a negative term. The registration error is applied for the positive term (experimentally obtained as r=1.95 pixel), while the halo effect forms the negative term, implying an opposite influence (h=5 pixel).

(2) $$G_{\sigma_{1}}(||p-q||)=exp\left(-\frac{||p-{q||}^{2}}{2\sigma_{1}^{2}}\right)$$

(3) $$\sigma_{1}=r-h$$

$G_{\sigma_{2}}$ is a univariate Gaussian distribution which represents the proposed thermal range similarity kernel (see Equation (4)). $\sigma_{2}$ determines the smoothness of the influence of $G_{\sigma_{2}}$. It can be calculated as in Equation (5), where H(V) is the information entropy of image V in grayscale, B is the bit depth of the visual image, and $p_{V}\left(i\right)$ is the probability of a histogram bin from the visual image. $\sigma_{I}$ is the standard deviation of the initial detection region, and $D_{KL}$ is the Kullback–Leibler divergence between a distribution and a uniform distribution.

(4) $$G_{\sigma_{2}}\left(\right|T_{q}-\bar{T_{I}}\left|\right)=exp\left(-\frac{|T_{q}-\bar{T_{I}}{|}^{2}}{2\sigma_{2}^{2}}\right)$$

(5) $$\sigma_{2}=\frac{\sigma_{T_{I}}}{D_{KL}}$$

(6) $$\begin{array}{ccc} D_{KL} & = & \sum_{i}p_{V}\left(i\right)log_{2}\frac{p_{V}\left(i\right)}{1/2^{B}}\\ & = & \sum_{i}p_{V}\left(i\right)\left(log_{2}p_{V}\left(i\right)-log_{2}\frac{1}{2^{B}}\right)\\ & = & \sum_{i}p_{V}\left(i\right)log_{2}p_{V}\left(i\right)-log_{2}\frac{1}{2^{B}}\sum_{i}p_{V}\left(i\right)\\ & = & -H(p)+B \end{array}$$

**Figure 4:**
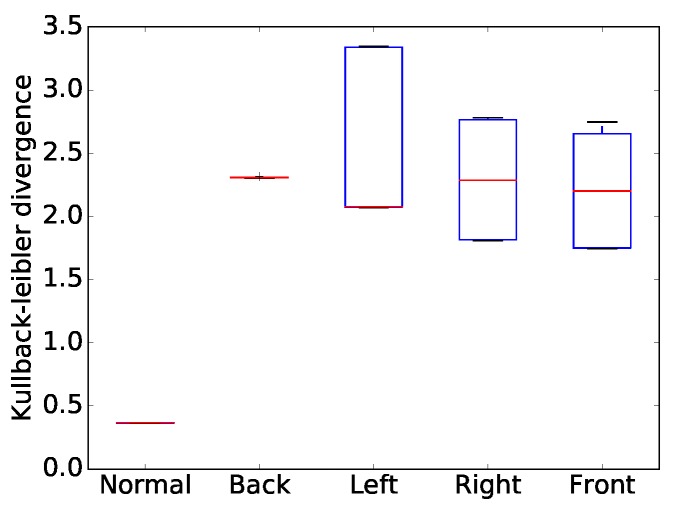
Kullback–Leibler divergence compared to uniform distribution in various illumination environments.

When a temperature value is close to the average temperature or the variation in temperature values is large, the response of the thermal range similarity kernel is large. Additionally, when the Kullback–Leibler divergence of a visual image, $D_{KL}$, is small, the response of the thermal range similarity kernel is large (as shown in Equation (7)). $D_{KL}$ [33] represents the degree of uniformity of a visual image (i.e., the amount of information). We address the issue of estimating the degree of uniformity of a visual image by calculating the similarity of a histogram distribution to a uniform distribution. We adopt the Kullback–Leibler divergence between an intensity distribution of visual image and a uniform distribution to build the measure of the degree of uniformity. A large Kullback–Leibler divergence means a small structural similarity to uniformity. A large amount of information is calculated when a visual image’s distribution resembles a uniform distribution. In order to fully exploit the large amount of information in a visual image, we allow a larger interval to the thermal range, and vice versa. To substantiate of the use of $D_{KL}$, we present Figure 4. The Kullback–Leibler divergences of the dark lighting conditions (e.g., lightings from back, left, right, and front sides) are relatively higher than that of the bright lighting condition. Eventually, the thermal range kernel is more sensitive for dark lighting conditions.

Because different subjects have different hand temperatures, we refer to the average temperature of a hand from a portion of the initial detection region. The portion has been set to 25% of the initial detection region, which is described in the following Section 3.3.1.

**Figure 5:**
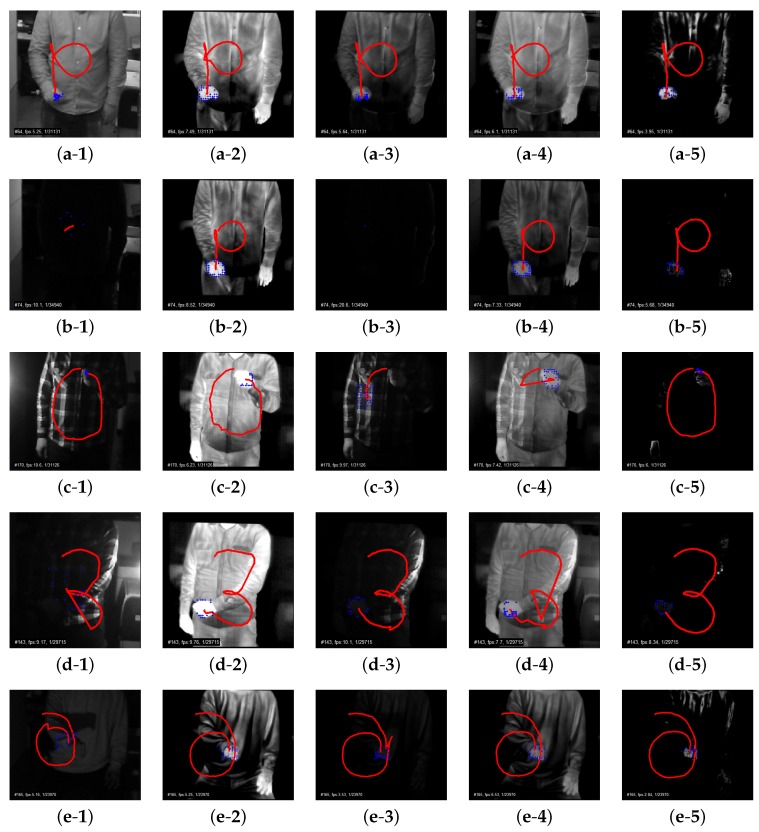
Example results of (**1**) *V* (Visual); (**2**) *T* (Thermal); (**3**) V*T (multiplication-based visual–thermal fusion method); (**4**) V+T (addition-based visual–thermal fusion method); and (**5**) V⨂T (joint filter-based method) in each lighting environment: (**a**) normal (bright); (**b**) back (dark); (**c**) left (dark); (**d**) right (dark); and (**e**) front (dark).

Firstly, Figure 5a shows the visual (*V*) and thermal (*T*) images and the corresponding results of three different representations in the bright (normal) lighting condition. One is the multiplication result (V*T, multiplication-based fusion method) between the visual and thermal images, another is the equally-weighted addition result (V+T), addition-based method) between the two images, and the other is the thermal-guided joint filter result (V⨂T, joint filter-based method) between the two modalities. In the presence of bright lighting, it is worth observing that the regions except the hand are effectively suppressed in V*T and V⨂T. The details of the texture of a hand are also preserved in V*T and V⨂T.

On the other hand, Figure 5b presents the results in the dark lighting condition from the back side. V*T no longer guarantees a meaningful compositeness in dark lighting conditions, whereas V⨂T effectively exploits the two modalities of thermal and visual information despite the dark lighting. As V+T computes an equally-weighted merger of the two images, the result is about the half-scaled intensity result of the thermal image in dark lighting conditions.

Additionally, Figure 5c–e provide the results in the dark lighting condition from left, right, and front sides, respectively. The tendency follows the combination of the influences of the bright lighting and the dark lighting from the back side. V⨂T better maintains the detailed texture of a hand compared to V*T and V+T, and better suppresses the regions besides the hand. The different aspect among left, right, and front side lighting conditions is that a hand is partially shown in left and right side lightings in the visual image, and the common aspect is that the three lighting conditions share similar representation in the thermal image.

### 3.3. Deep Learning-Based Hand Gesture Recognition

#### 3.3.1. Hand Gesture Tracking

We employed Tracking-Learning-Detection (TLD) [34] for the hand gesture tracking method. As proposed by Kalal et al. [34], TLD consists of three different phases: tracking, detection, and learning. The tracking part of the algorithm follows the object from frame to frame. The detection part localizes the observed appearances and corrects the tracking part. The learning part estimates the detecting part’s errors and updates the algorithm to avoid the same possible errors. The algorithm takes advantage of the initial detection region as a given initialization. Mostly, the default parameters of the author have been adopted, except for a smaller value of the minimum size of the tracking region and a smaller variance for the Gaussian kernel in the ensemble classifier for the detection part. The thermal-guided joint filter effectively and adjunctly consolidates the tracking scheme of TLD by providing better representation of a hand region. Based on the thermal-guided joint filter, tracking a hand becomes more stable by making the best use of both the detailed texture of a visual image and the meaningful cue of temperature from a thermal image. TLD operates without any prior training, but with an initially given detection region as a feature-based approach rather than an intensity-based one. As such, different hand temperatures can be considered as a minor hindrance to TLD-based tracking. That is one vital reason why we adopt TLD to track a hand gesture. Our proposed thermal-guided joint filter improves the feature representation of a hand when using heterogeneous sensors, and thereby enhances the robustness of the tracking performance against various lighting conditions.

#### 3.3.2. Hand Gesture Recognition

**Figure 6:**
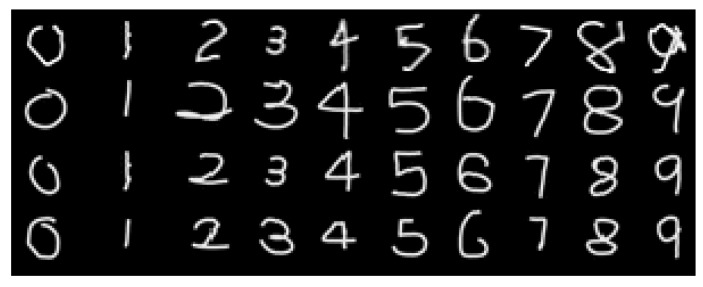
Examples of in-air-written digit number images of the thermo-visually calibrated (TVC)-hand gesture database.

**Figure 7:**
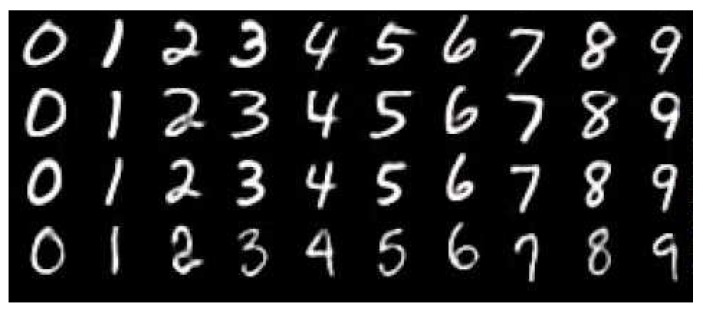
Examples of handwritten digit number images of the MNIST handwritten digits [44] database.

Classifying a handwritten digit becomes a more challenging problem in the context of a complex natural situation (e.g., writing a digit in the air using a hand gesture). Starting from hand-crafted features or template-matching, many recent studies leverage ConvNets [35] , which learn necessary features all the way from pixels to a classifier . Sermanet et al. [35] used the multi-stage features by branching out the outputs of every stage into the classifier and the Lp pooling approach to augment the traditional ConvNets architecture. The input of the classifier is an image obtained by accumulating the center points of a hand over multiple frames, as can be seen in Figure 6 (refer to [36]). As the dataset image sequence has been cropped when building the database to make the images sized 200×200 and centered at the center of trajectory, the sizes of digit examples can differ slightly from different subjects. However, the slight difference has little influence on the classification, as explained in Section 4. The input image for the classifier is transformed by accumulating the center points of a hand over multiple frames, as can be seen in Figure 6. Digit gesture—which has frequent changes in moving direction—is relatively more complicated than the gesture using triangles, rectangles, circles, etc. [37,38,39]. Moreover, digit gesture has been widely used to evaluate the recognition performance of an algorithm [36,40,41,42,43]. In this paper, we performed digit gesture classification by several classifiers (convolutional neural network (*CNN*) , Random Forests, and support vector machine (SVM)) based on hand gesture tracking, as we mainly focus on the potential feasibility of our proposed thermal-guided joint filter. As the purpose of adopting ConvNets is to verify the improvement in recognition performance based on our proposed method, the MNIST handwritten digits [44] (see Figure 7) database, as well as our thermo-visually calibrated-hand gesture (TVC-hand gesture) database have been adopted for this experiment (see Section 4.1.2). To deal with left-handed and right-handed subjects, we originally considered flipping the input image horizontally as a set of two mirrored images. Then, the one with a higher decision score was chosen for classification. Table 1 presents the operation and feature of our convolutional neural networks-based classifier which is implemented based on TensorFlow [45] using the ConvNet/SS model [46]. The classifier for hand gesture recognition is taught by using the *CNN* training tool of TensorFlow library. The TensorFlow library automatically provides an efficient backpropagation algorithm, and AdamOptimizer is used to modify the variables and minimize the loss as cross-entropy. In the Feature column in Table 1, 32@14×14 indicates that the convolution structure consists of 32 feature maps of 14×14 units, while 64@7×7 means that the convolution structure consists of 64 feature maps of 7×7 units. The stopping criterion of the training iteration has been set to 99.33% of classification performance in the training set, and the batch size for a training process is 500.

### 3.4. Overview

**Figure 8:**
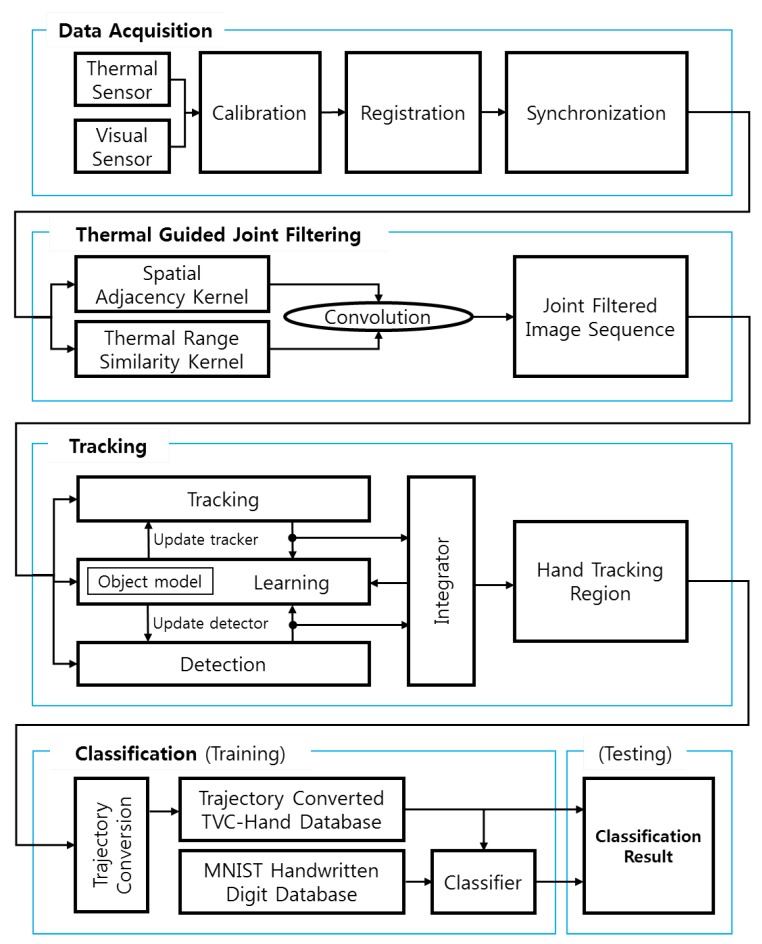
Overview of systematic flow.

Our experiment can be organized as the following steps, as shown in Figure 8.

- Step 1: Build the database for tracking and classifying a hand gesture as stated in Section 3.1.
  - Step 1-1: Calibration between the thermal and visual sensors
  - Step 1-2: Registration between the thermal and visual sensors
  - Step 1-3: Synchronization between the thermal and visual sensors
- Step 2: Compute the output image based on thermal-guided joint filter as stated in Section 3.2.
  - Step 2-1: Computation of spatial adjacency kernel
  - Step 2-2: Computation of thermal range similarity kernel
  - Step 2-3: Joint filtering based on the convolution using the two kernels
- Step 3: Track a hand gesture as stated in Section 3.3.1.
  - Step 3-1: Tracking phase
  - Step 3-2: Detection phase
  - Step 3-3: Learning phase
- Step 4: Classify the hand gesture as stated in Section 3.3.2.
  - Step 4-1: Training phase
  - Step 4-2: Testing phase

## 4. Experiments

### 4.1. Experimental Environment

#### 4.1.1. Hardware and Software

The combination of a visual camera and a thermal camera has been leveraged. We used a Microsoft Lifecam (960×544@30 Hz with wmv9 format) for the visual sensor and a FLIR S65 (320×240@30 Hz with 16 bits tiff format) for the thermal sensor. FLIR provides Equation (7) to convert raw signal values into meaningful temperature readings [47], where T is the temperature in Kelvin, RBFO are the external calibration parameterswhich are provided from meta data of the thermal video, and S is the 14-bit digital signal.

(7) T=B/log(R/(S−O)+F)

Matlab, Visual Studio, and TensorFlow in Python have been utilized for TLD, mex compiling, and recognition scheme, respectively. A desktop with Intel i7 960 (3.2 GHz, 12 GB memory) on Windows 10 has been utilized in our experiments.

#### 4.1.2. Database

**Figure 9:**
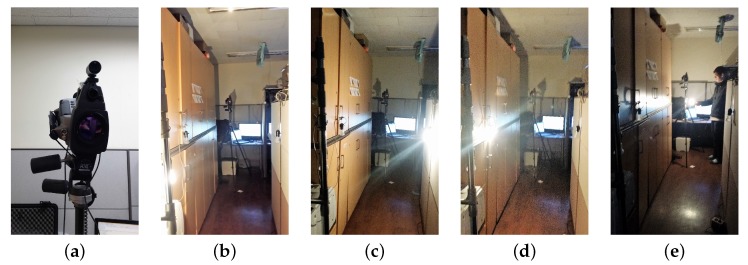
Configuration of data acquisition by a thermal camera and a visual camera, and different lighting conditions of the environmental configurations. (**a**) Configuration of data acquisition by a thermal camera and a visual camera; and the different environments of (**b**) back; (**c**) left; (**d**) right; and (**e**) front side lightings.

To remedy the deficiency in current works on thermo-visually calibrated data for hand gesture—especially for varying illumination environment—we collected 650 videos (image sequences) of hand gestures including ten different classes by a thermal camera and a visual camera to build a TVC-hand gesture database. The TVC-hand gesture database comprises a set of images with a checkerboard for calibration and a set of image sequences with hand gestures under various lightings. We started collecting data from a bright (normal) lighting environment and elaborated to dark lightings with four different partial lighting directions as shown Figure 9 (dark lighting conditions consist of back, left, right, and front side lightings). Four bulbs were used simultaneously as the sources of light and the sources of heat (both emitting light and radiating heat were considered). Thirteen subjects participated in data collection to provide gesture-written digits from 0 to 9. When building the database, each subject was asked to indicate his/her first and last frames in order to reflect his/her intention of a gesture (i.e., the information about starting and ending a gesture for each image sequence defines the gesture). Each gesture was conducted five times, as the gesture is captured once per lighting condition. A pair of visual and thermal image sequences is simultaneously captured to make integrated data for a hand gesture. For the registration of the two different modalities, we produced a checkerboard that performs the calibration both in the thermal image and the visual image as mentioned previously in Section 3.1. For the training phase of hand gesture recognition, MNIST and TVC-hand gesture databases were used together, whereas the TVC-hand gesture database was adopted to evaluate the performance of recognizing in-air written hand gestures. The TVC-hand gesture database provides the diversity of in-air written hand digits to the MNIST database, having a large quantity of handwritten digits. MNIST and TVC-hand gesture databases include 60,000 digit images and 650 hand gesture images (i.e., 130 hand gesture image sequences), respectively.

#### 4.1.3. Evaluation Metrics

By adopting the multi-class confusion matrix [48], the numbers of the four elements, true positive (TP), false positive (FP), true negative (TN), and false negative (FN), are obtained by one vs. all approach for each class. Then, true positive rate (TPR) and false positive rate (FPR) are calculated to draw a Receiver Operating Characteristic (ROC) [49,50] curve for experimental comparison. The ROC curve is plotted by measuring the number of true positives and the number of false positives at changing decision thresholds, which leads to a set of sequential true positive rates and false positive rates. Moreover, area under the curve (AUC) is used to visually compare the performances of all methods at a glance [51] (The Scikit-learn library [50] is leveraged to plot ROC curve and compute AUC). To avoid the overfitting problem, experiments are performed by five-fold cross-validation, where one-fold corresponds to the group for leave-one-group-out methodology. As the cross-validation is based on cross-subject scheme, one subject does not occur in different folds at the same time.

### 4.2. Experimental Results

#### 4.2.1. Bright Lighting Condition

The experimental results in the bright lighting condition show that all methods recognize hand gestures fairly well. The recognition using a single thermal image ranks the lowest among the compared methods. A sufficient amount of information has been included in the visual image compared to the thermal image. As this paper is putting emphasis on the experiment of the robustness against varying illumination, this normal lighting condition serves as a starting guideline.

#### 4.2.2. Dark Lighting Condition

##### Back Side Lighting

On the other hand, the experimental results in the dark lighting using back side lighting shows that the single visual image-based method suffers from the lack of visual information caused by the reduced overall brightness. The multiplication-based fusion method directly uses the visual information, so it behaves in the same way as with the single visual image-based method. The addition-based method consequently makes use of the thermal information in this lighting condition, leading to a similar result to that of the single thermal image-based method.

##### Left or Right Side Lighting

In partial lighting conditions such as left side lighting, the single thermal image-based method and the addition-based method show performance degradations. The physical distance between the right hand and the heat source located on the left side is relatively short (in the left lighting condition), and the heat source influences the temperature of the partial side of the hand. However, even though the partially emitted light source affects the visual appearance of a hand, the initial detection region itself starts with partially received lighting. The difference between the multiplication-based fusion method and the addition-based method comes from how the two methods respond to the horizontal error in registration. Specifically in the regions where the registration errors occur, the multiplication-based fusion method responds as the intersection calculation result between thermal and visual images, while the addition-based method responds as the union calculation result between the two images, which resembles an exaggerated halo effect.

##### Front Side Lighting

For the experiment in the presence of front side lighting condition, meaningful details of texture in the visual image still remain, despite the reduced overall brightness. Our proposed joint filter-based method and the addition-based method show slightly better performances than the single visual image-based method and the multiplication-based fusion method. The single thermal image-based method ranks the last, like in the normal lighting condition. Commonly, the partial lighting from front side causes generally diminished brightness in the visual image, but it provides a concentrated effect on a hand region, resulting in an increase in hand gesture recognition performance.

##### Comparing ROC Curves and AUCs

**Figure 10:**
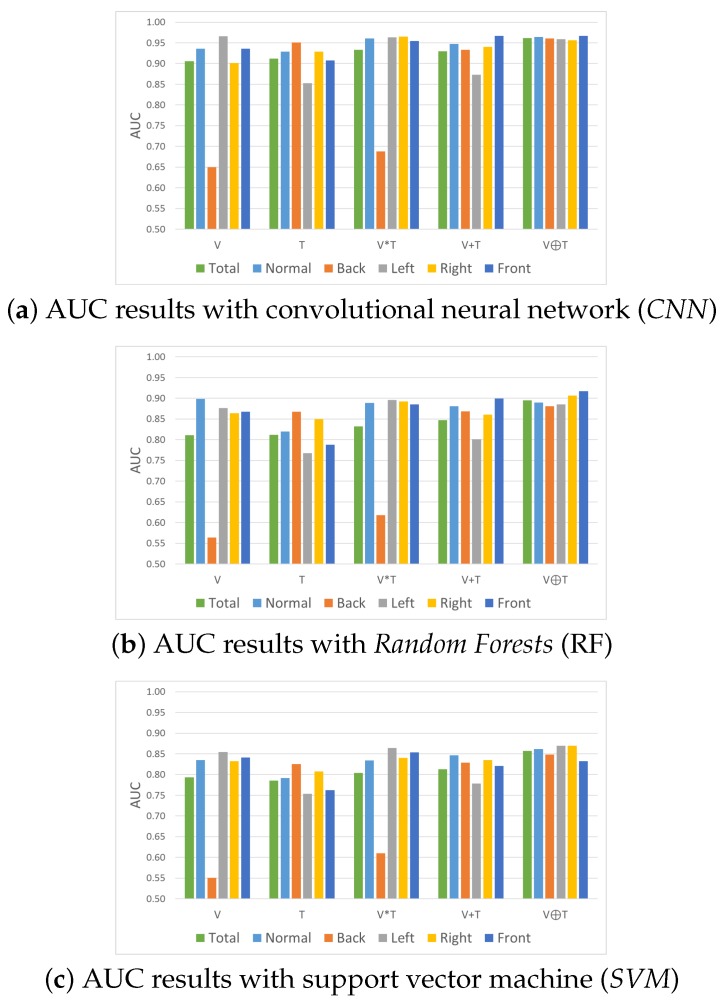
Area under the curve (AUC) comparison in various lighting conditions using (**a**) *CNN*; (**b**) RF; and (**c**) *SVM*.

**Figure 11:**
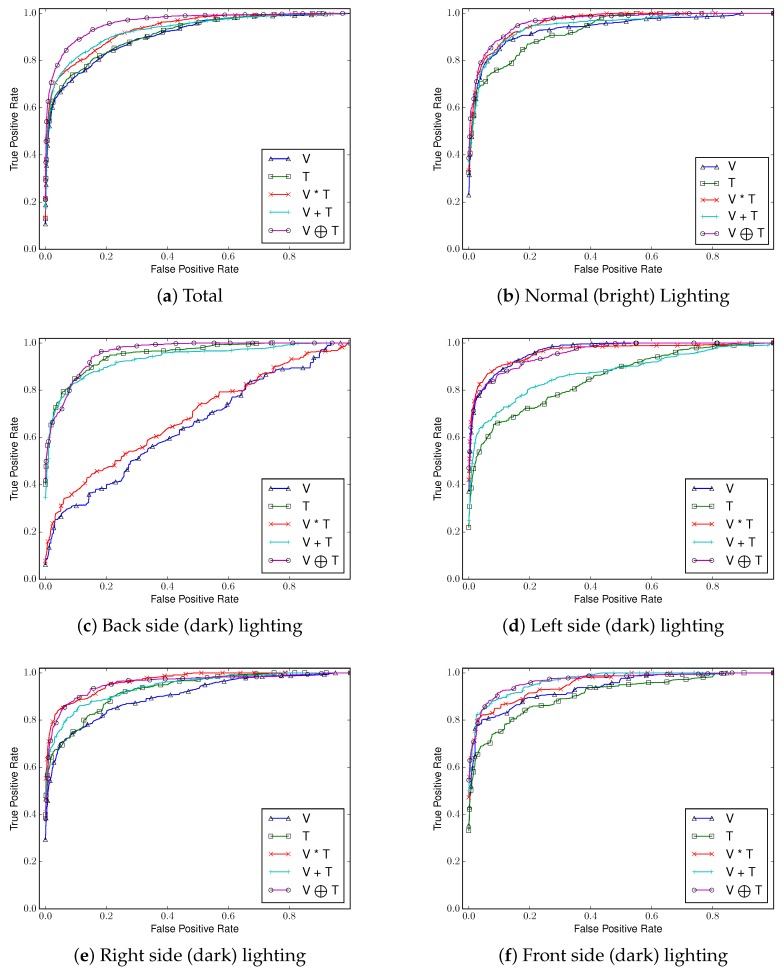
Receiver Operating Characteristic (ROC) curves of five compared methods in each lighting condition using *CNN*.

**Figure 12:**
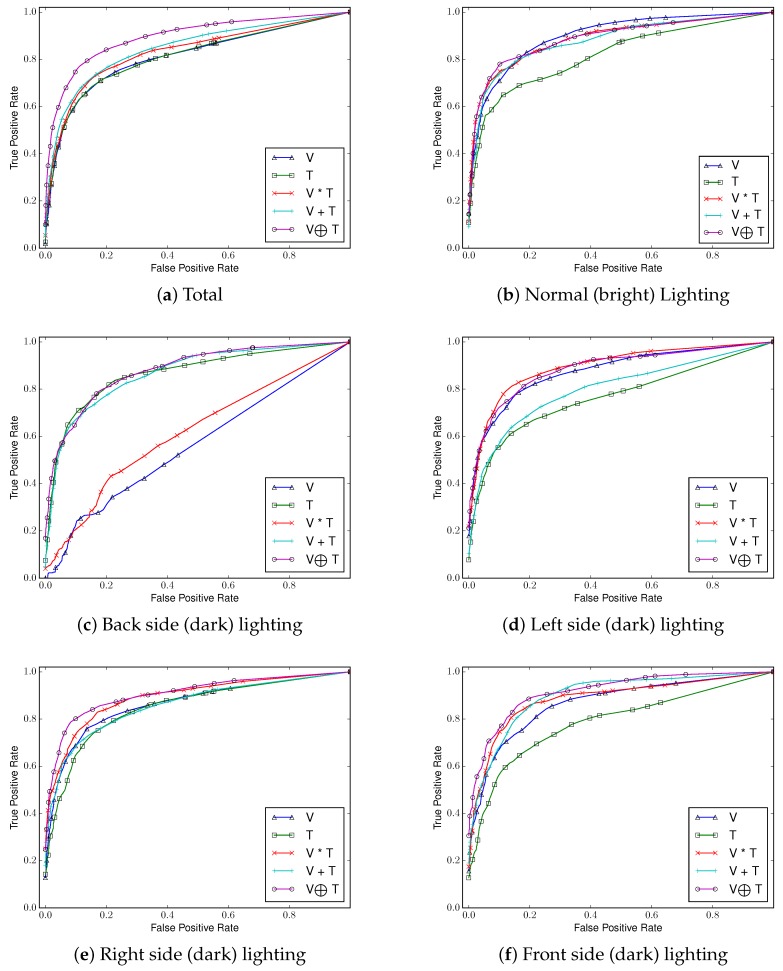
ROC curves of five compared methods in each lighting condition using *Random Forests*.

**Figure 13:**
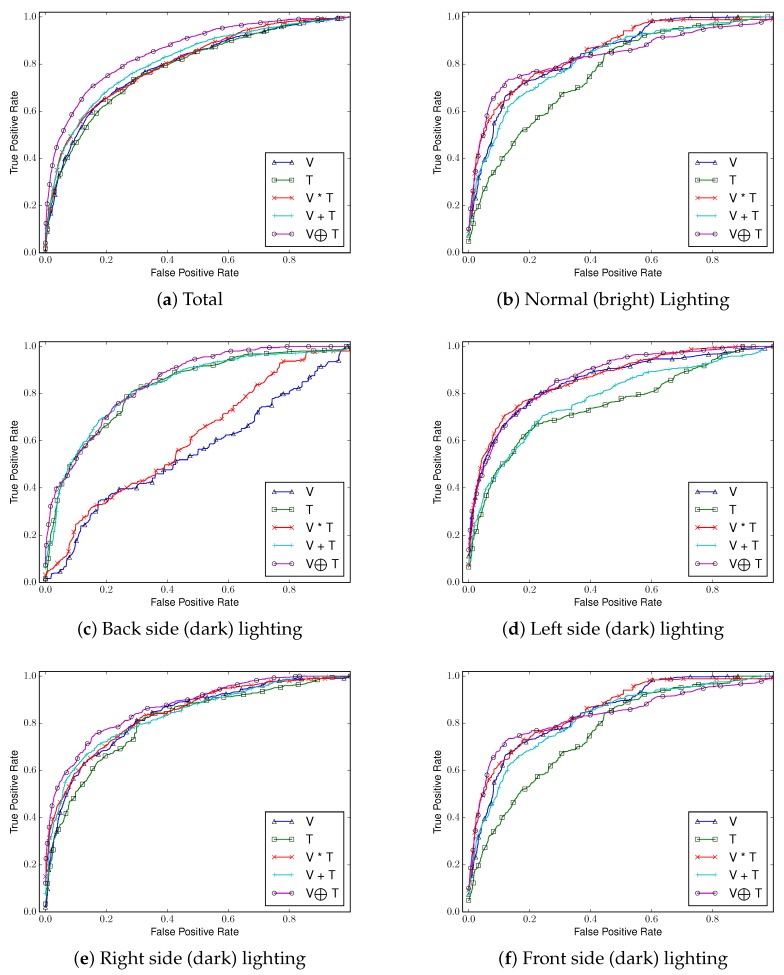
ROC curves of five compared methods in each lighting condition using *SVM*.

We use AUC and ROC curves for performance comparison as shown in Figure 10, Figure 11, Figure 12 and Figure 13. Considering the overall performance comparison, our proposed joint filter-based gesture recognition performs stably in the presence of varying lighting conditions compared to other methods (i.e., single visual image-based, single thermal image-based, multiplication-based, and addition-based methods). The darkest condition of back side lighting decisively influences the single visual image-based method and the multiplication-based fusion method. Another dark condition of left or right side lighting has a negative effect on the single thermal image-based method and the addition-based method. The results with all conditions (total) show that our proposed joint filter-based gesture recognition outperforms the other methods.

##### Computational Complexity

We measured the times for tracking and recognition, when recognition consists of training and testing. The average tracking processing time for a frame was compared, as each gesture has a different duration. Because of the two calculations of Gaussian distributions for the allowances of spatial adjacency and thermal range similarity, our proposed filter-based method consumes additional time, as presented in Table 2. However, we expect to further bridge the gap by code optimization or parallel computing. Considering that recognition has been used only as a means of verifying whether a hand gesture trajectory is well tracked, the compared methods share the identical training time and show similar testing time for recognition (as shown in Table 2).

##### Learning Data

**Figure 14:**
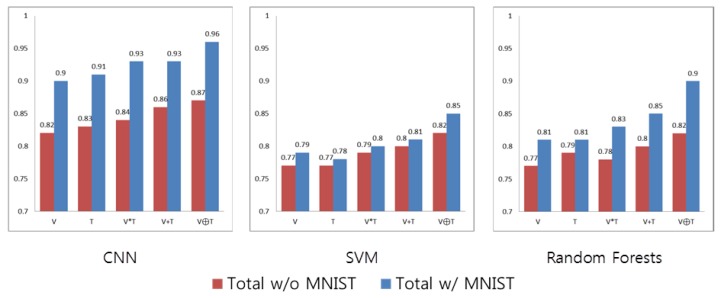
AUC comparison based on MNIST handwritten digits database usage.

In order to look into the influence of the comprehensive MNIST database, we repeat the previous experiments with exclusion of the MNIST learning data. As shown in Figure 14, the classification performances drastically decrease when using CNN, as a Neural Network-based classifier highly depends on data. On the other hand, SVM is based on particular data which correspond to support vectors, and the exclusion of the MNIST database has relatively less of an effect on the classification performance of SVM. Random Forests was influenced more than SVM by the learning data configuration. Accordingly, the large quantity of the MNIST database provides an improvement in the classification performance.

## 5. Discussion

It is possible for all tracking algorithms to fail to track the trajectory of a gesture, especially when the feature of a hand region is not sufficiently extracted because of a challenging lighting condition. That is the main reason why the recognition performances of the algorithms are all different, providing the comparative robustness against lighting conditions. Therefore, the effect on the recognition caused by the tracking failure of a gesture can be explained by the degradation of the recognition performance.

Thermal information provides the importance level of a hand region so as to enable selective exploitation of the visual content from informative regions. Visual information, on the other hand, not only takes part in determining the sensitivity of the thermal range kernel, but also compensates the insufficient details of a thermal image. Our experimental results verify that the performance of the hand gesture recognition is improved by adopting our proposed joint filter, compared to those of the single modality-based methods and those of simple fusion-based methods.

Compared to single modality-based methods, our method consistently represents a hand better, which leads to the improved performance in recognizing hand gestures. The thermal-guided joint filter makes use of adaptive portions of temperature values, which differ from different regions, based on the contribution of the two joint smoothness terms of spatial adjacency and thermal range similarity. Specifically, the thermal range similarity kernel provides an adaptive way to deal with different temperatures. An important note is that the information entropy of a visual region kernel internally determines the adaptiveness of thermal content. As a result, our proposed method not only performs hand gesture recognition effectively in bright lighting conditions, but also maintains the performance to a certain level in dark lighting conditions by preventing the environmental factor-caused performance degradation.

Comparing the methods of simple fusion, the hand gesture recognition using our proposed thermal-guided joint filter outperforms the multiplication-based fusion and addition-based fusion methods in the presence of various illumination conditions. The multiplication-based fusion method can suppress the influence of background which commonly has lower temperature than that of body heat in bright lighting condition. Yet, it shows drawbacks in dark lighting conditions, because the foreground unwillingly gets suppressed. With a partial light source (e.g., from left or right side) in dark lighting conditions, only the partial region on which the light is cast remains informative. On the other hand, the addition-based method generally involves the combined characteristics of thermal and visual images at the same time in the presence of bright lighting and the partial light source conditions. In dark lighting, the results of the addition-based method are akin to the thermal image itself. However, our proposed thermal-guided joint filter-based method highlights the detailed texture in the region where body heat emits relatively higher temperature in brightly lit regions, and also more or less conserves the detail in the dark region. Even for the partial lighting condition with left or right side lighting, our proposed method effectively works. In all conditions, it substantially suppresses the regions except for that of a hand. Eventually, our proposed method effectively recognizes hand gestures against varying illumination by its flexible exploit of thermal and visual contents.

Comparing the different classification methods, it is worth observing that CNN, Random Forests, and SVM generally show the similar tendency that the thermal guided joint filter provides strength against varying lighting conditions.

Another limitation is caused by registration error. Looking closer to the regions which contain the registration error, the multiplication-based fusion method returns the intersection calculation result between thermal and visual images without having much textural information, while the addition-based method returns the union calculation result between the two images, which resembles an exaggerated halo effect. On the other hand, the regions with the registration error tend to be blurred by the spatial adjacency kernel when using our joint filter. Along with further systematically minimizing the registration error between the two modalities, it is also possible for a theoretical development to be studied in our joint filter to alleviate this limitation.

## 6. Conclusions

We propose a new feature computing-based joint filter between thermal and visual information for hand gesture tracking and recognition. Visual images contain detailed texture, whereas they are vulnerable to varying lighting conditions. On the other hand, thermal images convey the meaningful cue of temperature, yet they have low resolution and a halo effect on edge regions. Thermal information has been studied in the literature to enrich visual information, but the existing approaches do not sufficiently utilize the cooperative relation between thermal and visual information in low feature level. Based on our method, the defects of the two modalities have been substantially complemented, and their merits have been fully exploited. In other words, our proposed method takes advantages of the thermal information’s stability against brightness and the visual information’s detailed texture, while complementing the low resolution and halo effect of thermal information and the visual information’s weakness against illumination. The feature of a hand gesture extracted by the proposed thermal-guided joint filter has been recognized by deep convolutional neural network. Our experimental results show that the performance of hand gesture recognition is stable against varying lighting conditions. Our method effectively recognizes hand gestures by consistently representing a hand in the presence of various lighting conditions. Based on the contribution of the joint smoothness terms of spatial adjacency and thermal range similarity, the thermal-guided joint filter provides flexible portions of temperature values differing from different regions. Still, reducing the computational time and minimizing registration error need to be studied.

For future work, we first plan to further investigate the benefit of the joint filter, based on Hidden-Markov Model (HMM) or Dynamic Time Warping (DTW) for the recognition and based on more complicated gestures and complex hand shapes. Then, we plan to improve the computation efficiency of our joint filter. As our method is easy to apply to other thermal-guided tasks, we also plan to apply our method to multiple sensor-based systems for recognition of body gesture or facial gesture, and to adopt the thermal-guided joint filter to different sensors.

## Figures and Tables

**Table 1:** The stage, operation, and feature of the classifier for hand gesture recognition. (ReLU means that the layer uses rectified linear unit as activation function).

Stage	Operation	Feature
1st stage	5×5 convolution with ReLU	32@14×14
2nd stage	5×5 convolution with ReLU	64@7×7
Densely connected stage	ReLU	1024
Decision stage	Softmax	10

**Table 2:** The average processing times for *CNN, Random Forests, and SVM*. Training and testing for recognition with *CNN* are performed with parallel processing by four-core CPU, and time per frame is measured for tracking. Unit: milliseconds.

Method			*V*	*T*	V*T	V+T	*V*⨂*T*
Tracking			150.1	159.3	155.4	172.9	222.7
Recognition	*CNN*	Training	11,316,850				
		Testing	9.39	9.38	9.35	9.58	9.43
	*Random Forests*	Training	205,833				
		Testing	186.55	186.77	185.97	185.91	186.77
	*SVM*	Training	3,085,456				
		Testing	127.99	128.37	128.60	128.36	128.47
